# Molecular Differences in Pancreatic Ductal Adenocarcinomas from Black versus White Patients

**DOI:** 10.1158/2767-9764.CRC-24-0376

**Published:** 2025-01-22

**Authors:** Saurabh Mandal, Emily A. Teslow, Minxuan Huang, Yingying Yu, Swathi Sridhar, Howard C. Crawford, Adam J. Hockenberry, Melissa C. Stoppler, Albert M. Levin, Ling Huang

**Affiliations:** 1 Henry Ford Pancreatic Cancer Center, Henry Ford Health, Detroit, Michigan.; 2 Tempus AI, Inc., Chicago, Illinois.; 3 Department of Pharmacology and Toxicology, Michigan State University, East Lansing, Michigan.; 4 Department of Public Health Sciences, Henry Ford Health, Detroit, Michigan.

## Abstract

Pancreatic cancer is the third leading cause of cancer-related death in the United States. Black or African American patients have a higher incidence of pancreatic cancer compared with other racial groups. It is unclear whether distinct molecular mechanisms are involved in the development of pancreatic cancer in different racial groups. To identify tumor molecular features that are distinctly associated with race in Black or African American and White patients with pancreatic ductal adenocarcinoma (the main subtype of pancreatic cancer), we analyzed deidentified patient records, including tumor sequencing data and expression of PD-L1, from the Tempus multimodal database. Patients with a primary diagnosis of pancreatic ductal adenocarcinoma and who received molecular testing between November 2017 and March 2023 were included in analyses. Among 4,249 patients analyzed in this study, 452 (10.6%) were Black or African American, and 3,797 (89.4%) were White. Black patients had a higher prevalence of *TP53* mutations compared with White patients (*P* < 0.001). *KRAS*^*G12R*^ mutations occurred more frequently in female patients in the Black versus White group (*P* = 0.007). Compared with White patients, Black patients had a higher tumor mutational burden (*P* < 0.001) and PD-L1 overexpression (*P* = 0.047). In a separate analysis of recent clinical trials testing immunotherapies for pancreatic cancer, we found that Black patients and other minorities were underrepresented in most trials. These findings suggest race-associated molecular differences in tumors that may impact patient responses to immunotherapies. Our study also supports the importance of improving patient diversity in clinical trials on pancreatic cancer treatments.

**Significance:**

By analyzing the records of patients with pancreatic cancer in the Tempus multimodal database, we identified genomic mutations and PD-L1 overexpression occurred more frequently in Black patients compared with their White counterparts. These molecular features may contribute to racial disparities in pancreatic cancer.

## Introduction

Pancreatic cancer ranks as the third leading cause of cancer-related deaths in the United States, with an estimated 64,050 new cases and 50,550 deaths in 2023 (National Cancer Institute). The current 5-year survival rate for pancreatic cancer stands at approximately 13%. Late diagnosis and lack of effective treatments with long-term effectiveness are significant contributors to the high mortality rates associated with pancreatic cancer (1). Another concerning trend is the continuous increase in new cases of pancreatic cancer in the United States over the past two decades (2). To tackle these challenges in pancreatic cancer care, there is an urgent need to enhance our understanding of this disease, ranging from uncovering novel molecular mechanisms of tumor–microenvironment interactions to delineating the roles of environmental factors and human genetic backgrounds in cancer initiation and progression.

An important observation from pancreatic cancer epidemiologic studies is the association between patient race with both disease occurrence and patient outcome. In a study analyzing data from the Surveillance, Epidemiology, and End Results (SEER) program, which collects cancer statistical data across the United States, Samaan and colleagues (2) reported that the Black or African American (BAA) population exhibits a higher age-adjusted incidence rate of pancreatic cancer than White individuals. This disparity is more pronounced among females than males. SEER data from 2000 to 2020 also revealed that BAA patients exhibit higher age-adjusted mortality rates and lower 5-year survival rates compared with White patients.

Analyses from multiple studies support that socioeconomic factors play a critical role in race-associated differences observed in the overall survival of patients with pancreatic cancer (3, 4). For example, Del Valle and colleagues (5), who examined data for patients with localized pancreatic cancer who underwent surgery at Veterans Health Administration (VHA) hospitals from 2006 to 2017, found no association between race and survival outcomes, after adjusting for socioeconomic status. Khalaf and colleagues (6) analyzed data for patients with pancreatic cancer at all disease stages in the VHA from 2010 to 2018 and found no significant differences in overall survival by race after accounting for socioeconomic factors. However, disparities were observed in patients with advanced-stage disease, in which BAA patients exhibited worse outcomes even after adjusting for other variables, based on analyses from the National Cancer Database (7).

In addition, there were notable race-associated variations in patient responses to specific treatments. Fang and colleagues (8) reported worse survival outcomes among BAA patients with lymph node metastases following resection compared with their White counterparts within a single-center study setting. Irfan and colleagues (9) observed that survival benefits conferred by systemic therapy postsurgical resection were only evident among White (and not BAA) groups. Ogobuiro and colleagues (10) discovered significantly lower major pathologic responses among BAA patients undergoing resection compared with their White counterparts across seven high-volume centers. These results suggest that biological mechanisms may also contribute to racial disparities in pancreatic cancer.

Studies on different types of cancers have highlighted the potential biological mechanisms contributing to disparities in human tumor development (11), including gene expression regulation, RNA processing, epigenetic modifications, and immune signaling pathways. These pathways potentially play important roles in driving the higher rates of pancreatic cancer in the BAA group. Interestingly, a study by Biel and colleagues examined gene expression profiles of pancreatic tumors from 5 BAA and 11 White patients and identified elevated levels of *TSPAN8*, a gene known to play crucial roles in pancreatic cancer (12), in BAA patients (13). To further evaluate the impacts of biological pathways on racial disparities in pancreatic cancer, we first need to identify molecular features, such as oncogenic mutations or immune regulators, differentially associated with race in a large population of patients.

In this study, we investigated the correlation between the molecular characteristics of pancreatic tumors and race within a large patient cohort, as an initial step toward understanding which biological pathways may partially drive racial disparities.

## Materials and Methods

### Patient inclusion criteria from the Tempus database

We analyzed deidentified patient records with a primary diagnosis of pancreatic ductal adenocarcinoma (PDAC) from the Tempus multimodal database (samples sequenced between November 2017 and March 2023). Patients over 18 years old and diagnosed with PDAC were included. Both female and male patients with self-reported races, such as “White,” “Black,” or “African American,” were selected for analysis. This study was determined as nonhuman subjects research by the institutional review board at Henry Ford Health.

### Analyses of Tempus data

All patients underwent somatic NGS testing via a commercially available 595 to 648 gene DNA panel (Tempus xT; refs. 14, 15) that assesses somatic mutations [including single nucleotide variants (SNV), Indels, copy number variations, and select structural variations], microsatellite instability (MSI), and tumor mutational burden (TMB). Details on sample preparation and bioinformatic analyses have been previously published (14, 15). Clinical and demographic information (including race) was abstracted from clinical records (e.g., order forms, and patient records). Statistical comparisons between White and Black samples were made with either the *χ*^2^ or Fisher’s exact tests. FDR corrections were applied to pairwise comparisons. All statistical analyses were performed in R version 4.2. This study did not use randomization and blinding data.

PD-L1 status for applicable samples was predominantly determined by Tempus clinical testing with the 22C3 anti-PD-L1 antibody (Agilent), as previously described (16). Briefly, slides were scored by a pathologist using the tumor proportion score (TPS), calculated as the percentage of tumor cells with complete or partial membrane staining. Samples are scored as PD-L1 negative (TPS < 1%), PD-L1 low (1%–49%), or PD-L1 high (≥50%). This study has been determined as nonhuman subject research by the internal review board at Henry Ford Health.

### Analyses of AACR GENIE data

Version 15.1 of AACR Project GENIE data hosted on cBioportal (www.cbioportal.org) were used for analyses. To avoid duplicating data from the same patients, only patients with single samples (Number of Samplers per Patient = 1) were selected for analyses. Other selection criteria on cBioportal include Cancer Type Detailed = “Pancreatic Adenocarcinoma,” Primary Race = “White” or “Black,” Center = “MSK” or “DFCI,” and Gene Symbols = “*TP53*” or “*KRAS*”.

### Analyses of SEER data

Analyses of SEER data were performed using the SEER Explorer (https://seer.cancer.gov/statistics-network/explorer/). The data source was SEER Incidence Data, November 2023 Submission (1975–2021).

### Data availability

Data used in the research were collected in a real-world healthcare setting and are subject to controlled access for privacy and proprietary reasons. When possible, derived data supporting the findings of this study have been made available within the manuscript and its Supplementary Figures/Tables. Tempus may make access to further deidentified data available pending a signed data use agreement, with data provisioned via Tempus LENS. Inquiries should be addressed to LENS@tempus.com.

## Results

### Patient characteristics

To investigate the relationship between the specific molecular attributes of pancreatic tumors and race (extracted from clinical records, see “Materials and Methods”) among patients, we analyzed deidentified data from individuals diagnosed with PDAC within the Tempus multimodal database. Our focus was on comparing the tumor profiles of White and BAA individuals, as the latter group has the highest incidence of pancreatic cancer in the United States, whereas the former constitutes the largest racial group among patients with pancreatic cancer. A total of 4,249 records of patients with pancreatic cancer were examined. As depicted in Table 1, 3,797 (89.36%) patients were White, and 452 (10.63%) were BAA. At the time of diagnosis, the median age for White patients was 68 years (IQR, 61–74; range, 22–90), and for BAA patients, it was 66 years (IQR, 58–73; range, 31–89). In the White patient group, 54% were male and 46% were female, whereas in the BAA group, 46% were male and 54% were female. The ethnicity of approximately half of the patients was not reported. Among those with recorded ethnicity, 5% of White patients and 0.5% of BAA patients identified as Hispanic or Latino. The percentages of patients without documented disease stages were similar in White (27.2%) and BAA (28.5%) patients (*P* = 0.57, *χ*^2^ test). At the time of biopsy collection, 1,087 White and 119 BAA patients had received chemotherapies.

**Table 1:** Characteristics of patients included in this study. Numbers in the brackets represent percentages of patients in designated groups

Characteristic	Overall, *N* = 4,249	White, *N* = 3,797	BAA, *N* = 452	*P* value
Age at Diagnosis				<0.001
Median (Q1, Q3)	68 (60, 74)	68 (61, 74)	66 (58, 73)	
Min, max	22, 90	22, 90	31, 89	
Gender				<0.001
Male	2,266 (53%)	2,060 (54%)	206 (46%)	
Female	1,983 (47%)	1,737 (46%)	246 (54%)	
Ethnicity				0.002
Not Hispanic or Latino	1,932 (95%)	1,715 (95%)	217 (100%)	
Hispanic or Latino	92 (4.5%)	91 (5.0%)	1 (0.5%)	
Unknown	2,225	1,991	234	
Smoker status				0.3
Current/former smoker	1,779 (52%)	1,581 (52%)	198 (55%)	
Never smoker	1,619 (48%)	1,458 (48%)	161 (45%)	
Unknown	851	758	93	
Chemotherapy prior to biopsy collection				0.3
No	3,043 (72%)	2,710 (71%)	333 (74%)	
Yes	1,206 (28%)	1,087 (29%)	119 (26%)	

### Genomic alterations associated with patient race

All patients in our study underwent targeted DNA sequencing tests for a panel of cancer-related genes (Tempus xT, see “Materials and Methods” section for details). Out of the population of patients who underwent testing, approximately 58% of the total population (57% of Whites and 58% of BAA) had tumor-normal matched analysis for Tempus xT, which utilizes an individual’s germline DNA as a reference genome to improve the accuracy of somatic mutation calling as well as improving accuracy in determining MSI and TMB status from NGS data in racially and ethnically diverse populations. To efficiently analyze patients’ genomic data, we selected genes with mutations occurring in at least 1% of patients with pancreatic cancer detected by whole exome sequencing in a recent study (17). Out of the 65 genes selected, 33 were common cancer-related genes that were sequenced in the Tempus xT panel (Supplementary Fig. S1). Within our study cohort, the prevalence of mutations in these 33 genes ranged from <0.1% (*SRC*) to 74% (*KRAS*).

**Figure 1:**
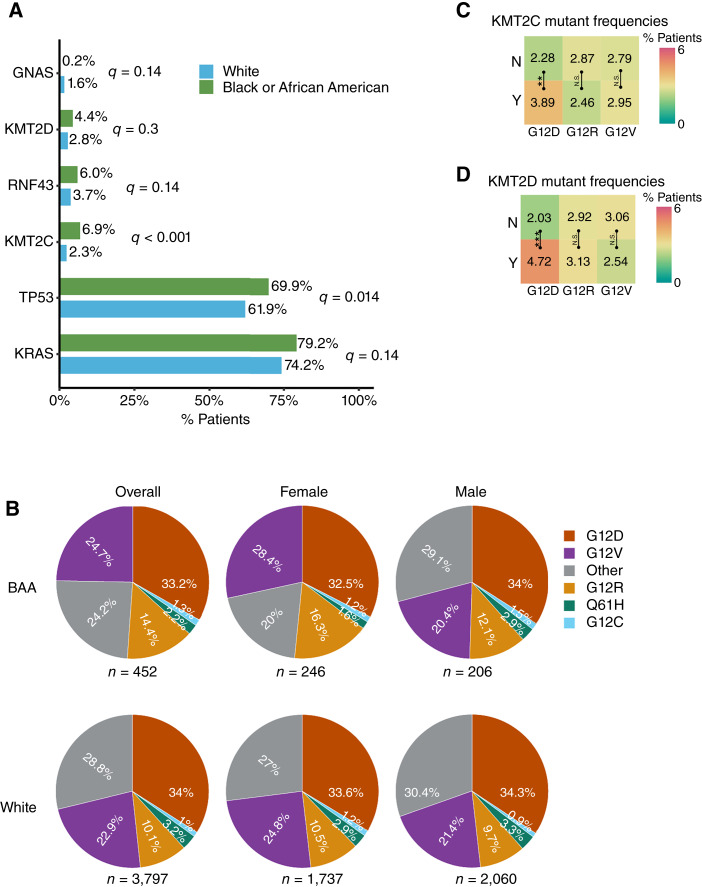
Genomic alterations in BAA and White patients with pancreatic ductal adenocarcinoma. **A,** Percentage of patients with genomic changes in specific genes (row name). *Q* values represent adjusted *P* values after FDR correction. **B,** Distributions of *KRAS* mutant alleles in patients with pancreatic cancer. The “other” group includes wild-type *KRAS* and all other mutations that are not G12D, G12V, G12R, Q61H, or G12C. **C** and **D,** Association between *KRAS* mutants and *KMT2C/D* mutants, respectively. The number and colors represent percentages of patients with *KMT2C* or *KMT2D* mutations in specific *KRAS* mutant groups (Y) and the rest of patients (N). Column names represent the specific *KRAS* mutant alleles analyzed. The significance of differences between groups was calculated by Fisher’s exact test or *χ*^2^ test. Significance representation: N.S., *P* ≥ 0.05; **, 0.001 ≤ *P* < 0.01; ***, *P* < 0.001.

Consistent with previous findings (18–20), *KRAS*, *TP53*, *CDKN2A*, *SMAD4*, *ARID1A*, *KDM6A*, and *RNF43* were among the most frequently mutated genes in tumors within our study cohort (Supplementary Table S1). Notably, we observed that for critical genes associated with pancreatic cancer development, such as *KRAS* and *TP53*, mutations occurred more frequently in the BAA group (Fig. 1A; *P* < 0.05). Other genes that exhibited a higher mutation prevalence in the BAA group included *KMT2D*, *RNF43*, and *KMT2C*. The likelihood of *KMT2C* mutations occurring in BAA patients was three times higher than in White patients. Conversely, *GNAS* was the sole gene with mutations detected significantly more commonly in the White group (Fig. 1A; *P* < 0.05). Interestingly, race-associated genomic mutational prevalences were influenced by patient sex as well. We further stratified patients by their sex and found that only *KMT2C* mutations were still significantly enriched in BAA patients across both genders (Supplementary Table S2). Among the female cohort, *TP53*, *KMT2D*, and *KRAS* mutations occurred more frequently in BAA patients compared with the White group (Supplementary Table S2). For male patients, *RNF43* mutations occurred more than three times more in the BAA than White group (Supplementary Table S2).

To determine whether race-associated differences in genomic alterations were significantly influenced by chemotherapies, we quantified gene mutation frequencies in patients who had no record of chemotherapy treatment prior to biopsy collection. Among this subcohort, we observed a similar trend: *KMT2C*, *KMT2D*, and *TP53* alterations were more prevalent in BAA patients, whereas White patients had higher frequencies of *GNAS* mutations (Supplementary Table S3).

To delve deeper into differences in genomic mutations by racial groups among patients with pancreatic cancer, we calculated frequencies of specific mutations within the two racial groups. Common *KRAS* mutations detected in pancreatic cancer included G12D, G12V, G12R, and Q61H. Only the G12R mutation had a significantly higher prevalence (*q* = 0.037) in the BAA group (14.4%) compared with the White group (10.1%; Fig. 1B). This race-associated disparity was even more pronounced among female patients, with *KRAS^G12R^* occurrence being 55% higher (*P* = 0.007) in BAA females than in White females (Fig. 1B). The mutant allele *KRAS*^*G12C*^, which is the target of several FDA-approved KRAS inhibitors, was identified in approximately 1% of the patients, with similar frequencies across the BAA and White groups. Specific *TP53* mutations (R273H, R175H, and R248Q) and *GNAS* (R201H and R201C) did not have significant differences in prevalence across the two racial groups (Supplementary Table S4).

Given our findings that *KMT2C* and *KMT2D* mutations were more prevalent in BAA patients and *KRAS^G12R^* was enriched in this racial group, we explored whether *KMT2C* and *KMT2D* mutations might be more frequently detected alongside certain *KRAS* mutant alleles. Among patients with *KRAS^G12D^* mutants, 3.89% had *KMT2C* mutations compared with 2.28% for *KMT2C* mutations in patients without *KRAS^G12D^* mutants (wild-type or other *KRAS* mutants; *P* < 0.01; Fig. 1C). Furthermore, *KMT2D* mutations occurred more frequently in patients with *KRAS^G12D^* alleles (*P* < 0.001; Fig. 1D). No associations between *KMT2C/D* and other *KRAS* mutant alleles were observed.

**Figure 2:**
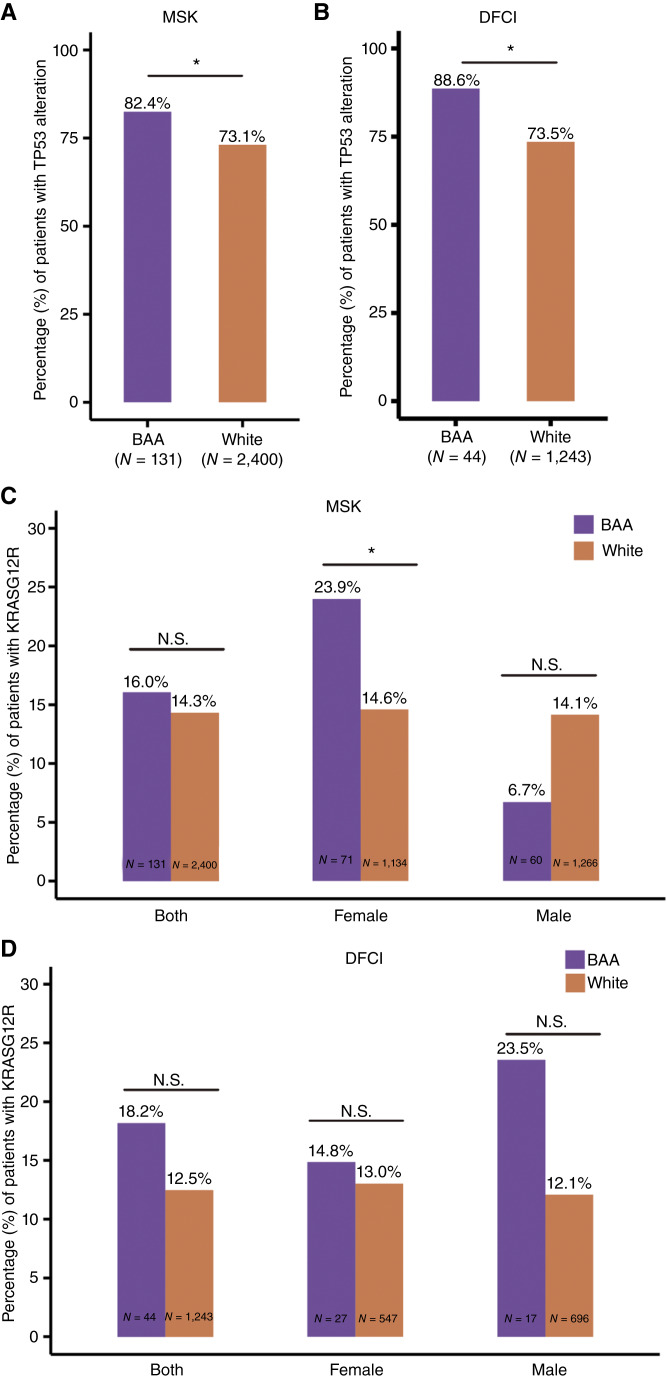
*TP53* and *KRAS* mutations in BAA and White patients from cohorts in AACR Project GENIE. **A** and **B,** frequencies of *TP53 *genomic alterations in BAA (purple) and White (orange) patients from MSK (**A**) and DFCI (**B**). **C** and **D,** frequencies of *KRAS*^*G12R*^ in BAA (purple) and White (orange) patients from Memorial Sloan Kettering Cancer Center (MSK, **C**) and (DFCI, **D**). *P* value indicators: N.S., *P* ≥ 0.05; *, 0.01 ≤ *P* < 0.05.

We next investigated whether the association between race and genomic features identified in our study was observed in other patient cohorts. AACR Project GENIE is a cancer registry of real-world clinic–genomic data from multiple institutions (21). Because participating institutions use different sequencing platforms and analysis pipelines, we focused on genomic alterations of *KRAS* and *TP53* (the top two altered genes in pancreatic cancer) in individual centers. Memorial Sloan Kettering Cancer Center (MSK) and Dana Farber Cancer Institute (DFCI) cohorts include more than 1,000 patients of interest for our study (White and BAA combined) and thus were used in our analyses. The frequencies of *TP53* alterations in BAA patients were significantly higher than in White patients, in both MSK (*P* = 0.024) and DFCI (*P* = 0.038) cohorts (Fig. 2A and B). We also detected a trend for *KRAS^G12R^* to occur more frequently in BAA female patients, particularly in the MSK cohort (*P* = 0.048; Fig. 2C and D). Those observations were consistent with patterns discovered in our primary patient cohort.

### TMB in patients of different races

**Figure 3:**
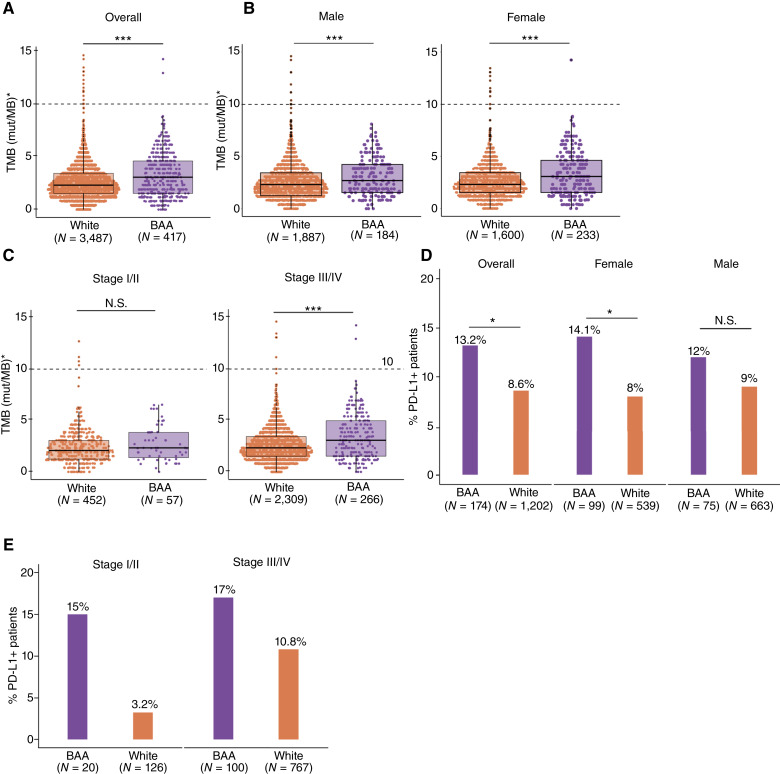
Cancer immunology-related molecular features of tumors from BAA and White patients. **A** and **B,** TMB of tumors from BAA (purple) and White (orange) patients. **B,** TMBs in patients stratified by race and sex. **C,** TMB of tumors from BAA (purple) and White (orange) patients at different clinical stages. **D,** Percentage of PD-L1 positive patients in BAA (purple) and White (orange) groups. **E,** Percentage of PD-L1 positive BAA (purple) and White (orange) patients at different clinical stages. The significance of differences between groups was calculated by Fisher’s exact test or *χ*^2^ test. Significance representation: N.S., *P* ≥ 0.05; *, 0.01 ≤ *P* < 0.05; ***, *P* < 0.001.

TMB measures the number of protein sequence–altering SNVs and indels per million coding base pairs. TMB serves as a potential marker for predicting responses to immunotherapies, based on the rationale that more neoantigens generated by increased mutations will enhance T-cell recognition probabilities. We quantitated the TMB of patients’ tumors based on the Tempus xT panel sequencing. The median TMB across all patients was 2.34 mut/Mb (IQR, 1.53–3.43; Fig. 3A; Supplementary Table S5). BAA patients exhibited a significantly higher TMB with a median value of 3.07 mut/Mb compared with White patients with a median TMB of 2.31 mut/Mb (*P* < 0.001). This race-associated difference was consistent in both female and male patients (Fig. 3B). To further explore whether TMB differences between BAA and White patients were influenced by disease stage progression, we conducted TMB comparisons among patients stratified by stage. In early-stage patients (stage I/II), BAA and White patients displayed comparable TMB levels; however, among late-stage patients (stage III/IV), which represented >70% of the total cohort analyzed in this study, BAA patients demonstrated significantly higher TMB levels (Fig. 3C; *P* < 0.001).

### PD-L1 expression in patients of different races

Evading immune surveillance is a hallmark of cancer. The PD-1/PD-L1 immune checkpoint molecules are frequently overexpressed during tumor progression and are common targets for cancer immunotherapies. Among our study participants, PD-L1 expression was evaluated by IHC in 1,376 individuals. Overall, 9.2% of patients tested positive for PD-L1 expression. Notably, a higher proportion of BAA patients (13.2%) tested positive for PD-L1 compared with White patients (8.6%; Fig. 3D; Supplementary Table S5; *P* = 0.047). This discrepancy was more pronounced among female individuals than males (Fig. 3D). Surprisingly, this race-associated difference was also evident even among early-stage patients: Although PD-L1 positivity was observed in 15% of BAA stage I/II patients, only 3.2% of White stage I/II patients exhibited PD-L1 positivity (Fig. 3E). A similar trend was also observed in stage III/IV patients. Disparities between stage-stratified patient groups did not reach statistical significance likely due to insufficient numbers of individuals with both PD-L1 scores and stage information.

### BAA patient enrollment in pancreatic cancer immunotherapy trials

The observation that PD-L1 overexpression is more prevalent among BAA patients suggested that racial background could influence immune suppression during pancreatic cancer progression, which may lead to variations in responses to immunotherapy across different races. Previous studies have highlighted the underrepresentation of minority groups in oncology clinical trials. To assess the representation of BAA individuals in recent clinical trials focusing on immunotherapies for pancreatic cancer treatments, we conducted a literature survey using PubMed to identify clinical trials published between 2018 and 2024 encompassing immunotherapy arms targeting pancreatic cancer. Out of the 32 reports examined, 15 reports enrolled at least 10 participants and included self-reported race data within patient demographics (refer to Table 2; refs. 22–36). The drugs tested across these studies included CD40 agonists, PD-1 or PD-L1 inhibitors, and CTLA-4 inhibitors. Most studies were classified as phase I or II trials. Those studies predominantly enrolled White participants at an average percentage of approximately 89.7%. The enrollment percentage for BAA participants ranged from 0 to 8% across these studies with an average representation standing at merely 3.8%. This is notably lower than the proportion of BAA individuals within the overall US population as per the data from the latest census conducted in 2020, which reported approximately 13.6%. Other minority groups such as Asian Americans as well as American Indian/Native Hawaiian/Pacific Islanders also exhibited underrepresentation within these clinical trials.

**Table 2:** Summary of race representation in clinical trials evaluating immunotherapies for pancreatic cancer. Numbers in the brackets represent percentages of patients in designated groups

Study	Trial number	Total number of patients	White (%)	BAA (%)	Asian (%)	American Indian/Native (%)	Native Hawaiian/ Pacific Islander (%)	Other (%)	Immunotherapy drug
Byrne and colleagues (22)	NCT02588443	16	15 (94)	0 (0)	0 (0)	0 (0)	0 (0)	1 (6)	Selicrelumab
Hewitt and colleagues (23)	NCT01836432	303	275 (91)	17 (6)	5 (2)	0 (0)	0 (0)	6 (2)	HAPa
Kamath and colleagues (24)	NCT01473940	21	20 (95)	1 (5)	0 (0)	0 (0)	0 (0)	0 (0)	Ipilimumab
Katz and colleagues (25)	NCT02305186	37	34 (92)	1 (3)	0 (0)	0 (0)	0 (0)	2 (5)	Pembrolizumab
Leidner and colleagues (26)	NCT02452268	83	74 (89)	3 (4)	4 (5)	0 (0)	0 (0)	2 (2)	NIZ985
Lin and colleagues (27)	NCT01959672	11	10 (91)	0 (0)	0 (0)	0 (0)	0 (0)	1 (9)	Oregovomab
O'Hara and colleagues (28)	NCT03214250	24	21 (88)	2 (8)	1 (4)	0 (0)	0 (0)	0 (0)	Sotigalimab
O’Neil and colleagues (29)	NCT03080974	10	9 (90)	0 (0)	0 (0)	0 (0)	0 (0)	1 (10)	Nivolumab
Overman and colleagues (30)	NCT02362048	73	62 (85)	4 (5)	2 (3)	0 (0)	0 (0)	5 (7)	Pembrolizumab
Padron and colleagues (31)	NCT03214250	105	88 (84)	5 (5)	7 (7)	0 (0)	0 (0)	5 (5)	Nivolumab,sotigalimab
Parikh and colleagues (32)	NCT03104439	25	23 (92)	0 (0)	0 (0)	0 (0)	0 (0)	2 (8)	Ipilimumab, nivolumab
Reiss and colleagues (33)	NCT03404960	84	75 (89)	6 (7)	2 (2)	0 (0)	0 (0)	1 (1)	Nivolumab, ipilimumab
Rojas and colleagues (34)	NCT04161755	19	19 (100)	0 (0)	0 (0)	0 (0)	0 (0)	0 (0)	Atezolizumab
Wang–Gillam and colleagues (35)	NCT02546531	42	36 (86)	3 (7)	0 (0)	0 (0)	0 (0)	3 (7)	Pembrolizumab
Zamarin and colleagues (36)	NCT02301130	64	52 (81)	5 (8)	4 (6)	0 (0)	0 (0)	3 (5)	Mogamulizumab, durvalumab

## Discussion

The number of patients newly diagnosed with pancreatic cancer in the United States has continued to rise in the last decade. There are significant disparities in pancreatic cancer incidence among racial groups, with the BAA population being the most affected. In this study, we analyzed genomic alterations and immune checkpoint biomarker expression in one of the largest and most racially diverse cohorts of patients with pancreatic cancer. We found that several cancer-related genes, such as *KRAS*, *TP53*, and *KMT2C*, were more frequently altered in BAA patients. PD-L1 overexpression was also more prevalent in BAA individuals compared with the White group. These molecular variances, along with discrepancies in socioeconomic status and environmental factors, may be responsible for the higher incidence of pancreatic cancer in the BAA group compared with the general population.

Previous studies have suggested that socioeconomic factors play a central role in race-associated differences in the overall survival of patients with pancreatic cancer. It is important to note that the current standard of care includes surgery and chemotherapies (such as gemcitabine/nab-paclitaxel or FOLFIRINOX), and no targeted therapy has been widely used in pancreatic cancer treatments. As novel treatment options like KRAS inhibition or immunotherapies undergo evaluation for efficacy against pancreatic cancer, it will be intriguing to see whether differential responses based on molecular features distinctly associated with race may emerge toward therapeutics targeting specific biological domains.

*KRAS* and *TP53* are the two most mutated genes in pancreatic cancer. Previous research has shown that mutations in *KRAS* and *TP53* are adequate to induce the formation of PDAC in animal models (37). Mutations in *KRAS* and *TP53* impact cell proliferation, genomic stability, and other cellular activities. Interestingly, increased genomic alterations of *TP53* have been reported in BAA groups across various cancers such as breast cancer, endometrial cancer, and gastric cancer (38–42). Higher frequencies of *KRAS* mutations have also been observed in colon cancer (43–45).

Apart from an overall increase in *KRAS* mutations, we also noted a specific *KRAS* mutant allele, *KRAS^G12R^*, which was significantly more prevalent in BAA patients with pancreatic cancer. This mutant allele is found at high frequencies (>10%) only in pancreatic cancer, but not in colon or lung cancer patients. Biochemically, *KRAS^G12R^* is deficient in activating the PI3K-AKT pathway (46), and targeting this mutation in pancreatic cancer may require strategies that differ from those used to inhibit *KRAS^G12D^*and *KRAS^G12V^*. Associations between specific *KRAS* mutant alleles and race have been discovered in other cancer types. For instance, Wang and colleagues (47) reported that White patients with lung cancer had higher frequencies of *KRAS^G12C^* mutants compared with Asian patients. They also showed that among the White patients, increased portions of genetically inferred European ancestry were linked to a higher likelihood of *KRAS* mutations. Self-reported race is a social construct, whereas genetically inferred ancestry is a biologically based estimate of an individual’s ancestry (48). Our study only assessed racial differences but further delving into ancestry-based differences represents a promising area for future research, as these two related but different classifications can be complementary in helping to uncover the socioeconomic and biological mechanisms underlying racial disparities in cancer.

The upregulation of immune checkpoint molecules, such as PD-L1 or PD1, is common during cancer development and serves as a critical mechanism for suppressing immune responses in the tumor microenvironment. In our study, we observed that PD-L1 overexpression was more prevalent in BAA patients compared with White patients with pancreatic cancer, particularly in stage I/II patients. This finding suggests that there may be race-associated differences in biological pathways that regulate immunologic responses during cancer development, potentially contributing to the high incidence rates of pancreatic cancer in the BAA population. The association between higher PD-L1 expression and African heritage has also been reported in triple-negative breast cancer and prostate cancer (49–51), indicating that the upregulation of immune checkpoint molecules may be a common biological pathway that increases cancer incidences among individuals of African descent.

Although current immunotherapy strategies have not yet significantly improved patient survival in pancreatic cancer, there is potential for new immunologic agents to effectively inhibit pancreatic tumor progression in the future. It is crucial to include a diverse population of patients in clinical trials for pancreatic cancer care, especially considering that these patients may differentially benefit from such interventions. In fact, race-associated differences in responses to immunotherapies have been observed in prostate cancer: African American patients who received the Sipuleucel-T cancer vaccine responded significantly better compared with White patients (median overall survival 35.3 months versus 25.8 months in a PSA-matched set; ref. 52). Our analysis of patient demographics enrolled in immunotherapy trials for pancreatic cancer revealed that BAA and other minority groups were underrepresented in these trials, consistent with the broader trend of historically disadvantaged groups being underrepresented in oncology clinical trials (53–57). From a cancer biology perspective, our findings underscore the importance of including a diverse patient population in clinical trials for pancreatic cancer treatments to develop therapies that can benefit all patients and address racial disparities in pancreatic cancer care.

One interesting observation in our analyses is that the association between race and molecular features of tumors from patients with PDAC was influenced by sex. Interactions between race, sex, and genomic alterations have been reported in lung cancer and colorectal cancer (58, 59). Biological pathways that contribute to sex differences in cancers of nonreproductive organs include epigenetics, metabolisms, and immunity (60, 61). A promising area of future research may be to investigate how those pathways may differentially regulate the biology of pancreatic cancer in different racial and sex groups.

In our study cohort, among patients with recorded disease stages, 76% had stage 4 cancer at the time of testing, a figure higher than the overall proportion of stage 4 patients in the United States (SEER). This trend was consistent among both White and BAA patients in our study, possibly indicating that healthcare providers are more inclined to order molecular testing for late-stage patients to explore additional treatments based on genomic mutations. In addition, more than half of the patients with smoking history available in both White and BAA groups were current or former smokers, a significantly higher proportion compared with the general adult population in the United States (11.5%; ref. 62).

To conclude, we examined molecular characteristics of pancreatic tumor tissues from BAA and White patients and identified genomic alterations and immune checkpoint molecular expression associated with BAA individuals. Our findings strongly support that the frequency of somatic tumor mutations differs in BAA patients, which may contribute to racial disparities in pancreatic cancer mortality. This study emphasizes the need for further research to elucidate signaling pathways influencing pancreatic cancer development among individuals of different races and underscores the urgency of increasing diversity among patients included in clinical trials for pancreatic cancer.

### Strengths and limitations

Our study analyzed one of the largest and most racially diverse cohorts of patients with PDAC. We identified tumor genomic mutations and an immunosuppressive marker in the tumor microenvironment associated with patient races. These findings provide clinically based evidence supporting the role of biological factors in racial disparities related to pancreatic cancer development. In our study, the prevalence of select genomic alterations was lower than what has been reported in several other studies. Our data are derived from clinical samples, and patients are more likely either at higher risk or later stage. However, this consisted of a very heterogeneous population of patients, 24% of which were at an earlier stage. The difference in prevalence between this cohort of patients and other previously published reports is currently not well understood. We emphasize that all patients were subject to the same analysis pipeline; therefore, the comparisons between races should not be affected by this factor. We also acknowledge that DNA was extracted from FFPE slides provided by different clinical sites, and some operative factors were not possible to control for. Our study focused on a panel of oncogenes, which were selected based on the results of previous studies in which patients were predominantly White. Future studies that compare whole genome sequencing data of PDAC tumors from BAA and White patients will provide more details on race-associated genomic alterations in PDAC and their influences on cancer disparities. In addition, TMBs of tumors were calculated based on exome sequencing of selected genes instead of whole genome sequencing. Conclusions from our study will need further validation by studies from other large databases.

## Supplementary Material

Supplementary Table S1 Genomic alterations of PDAC-associated genes in our study cohort.

Supplementary Table S2 Somatic mutational prevalences in PDAC patients stratified by their sex and race.

Supplementary Table S3 Somatic mutational frequencies in PDAC patients without chemotherapies at the time of biopsy.

Supplementary Table S4 Prevalences of specific mutant alleles in TP53 and GNAS genes in White and BAA patients with PDAC.

Supplementary Table S5 Tumor mutational burdens and PD-L1 expression in White and BAA patients.

## References

[1] Park W , Chawla A, O’Reilly EM. Pancreatic cancer: a review. JAMA 2021;326:851–62. 34547082 10.1001/jama.2021.13027 PMC9363152

[2] Samaan JS , Abboud Y, Oh J, Jiang Y, Watson R, Park K, . Pancreatic cancer incidence trends by race, ethnicity, age and sex in the United States: a population-based study, 2000–2018. Cancers (Basel) 2023;15:870. 36765827 10.3390/cancers15030870 PMC9913805

[3] Blanco BA , Poulson M, Kenzik KM, McAneny DB, Tseng JF, Sachs TE. The impact of residential segregation on pancreatic cancer diagnosis, treatment, and mortality. Ann Surg Oncol 2021;28:3147–55. 33135144 10.1245/s10434-020-09218-7

[4] Lee S , Reha JL, Tzeng C-W, Massarweh NN, Chang GJ, Hetz SP, . Race does not impact pancreatic cancer treatment and survival in an equal access federal health care system. Ann Surg Oncol 2013;20:4073–9. 24002535 10.1245/s10434-013-3130-3

[5] Del Valle JP , Fillmore NR, Molina G, Fairweather M, Wang J, Clancy TE, . Socioeconomic disparities in pancreas cancer resection and survival in the veterans health administration. Ann Surg Oncol 2022;29:3194–202. 35006509 10.1245/s10434-021-11250-0 PMC13171219

[6] Khalaf N , Xu A, Nguyen Wenker T, Kramer JR, Liu Y, Singh H, . The impact of race on pancreatic cancer treatment and survival in the nationwide veterans affairs healthcare system. Pancreas 2024;53:e27–33. 37967826 10.1097/MPA.0000000000002272 PMC10883640

[7] Jogerst K , Zhang C, Chang Y-H, Abujbarah S, Ali-Mucheru M, Pockaj B, . Socioeconomic and racial disparities in survival for patients with stage IV cancer. Am J Surg 2023;226:20–7. 36922322 10.1016/j.amjsurg.2023.03.003

[8] Fang HA , Irfan A, Vickers SM, Gbolahan O, Williams GR, Outlaw D, . Are lymph node metastases associated with survival in Black patients with pancreatic cancer? J Surg Res 2023;284:143–50. 36571869 10.1016/j.jss.2022.11.031

[9] Irfan A , Fang HA, Awad S, Alkashah A, Vickers SM, Gbolahan O, . Does race affect the long-term survival benefit of systemic therapy in pancreatic adenocarcinoma? Am J Surg 2022;224:955–8. 35430088 10.1016/j.amjsurg.2022.04.004

[10] Ogobuiro I , Collier AL, Khan K, de Castro Silva I, Kwon D, Wilson GC, . Racial disparity in pathologic response following neoadjuvant chemotherapy in resected pancreatic cancer: a multi-institutional analysis from the central pancreatic consortium. Ann Surg Oncol 2023;30:1485–94. 36316508 10.1245/s10434-022-12741-4 PMC13188206

[11] Freedman JA , Al Abo M, Allen TA, Piwarski SA, Wegermann K, Patierno SR. Biological aspects of cancer health disparities. Annu Rev Med 2021;72:229–41. 33502900 10.1146/annurev-med-070119-120305

[12] Li J , Chen X, Zhu L, Lao Z, Zhou T, Zang L, . SOX9 is a critical regulator of TSPAN8-mediated metastasis in pancreatic cancer. Oncogene 2021;40:4884–93. 34163029 10.1038/s41388-021-01864-9 PMC8321899

[13] Biel TG , Petrovskaya S, Mascia F, Ju T, Fashoyin-Aje L, Herremans KM, . Transcriptomic analysis of pancreatic adenocarcinoma specimens obtained from Black and White patients. PLoS One 2023;18:e0281182. 36812168 10.1371/journal.pone.0281182 PMC9946261

[14] Beaubier N , Bontrager M, Huether R, Igartua C, Lau D, Tell R, . Integrated genomic profiling expands clinical options for patients with cancer. Nat Biotechnol 2019;37:1351–60. 31570899 10.1038/s41587-019-0259-z

[15] Beaubier N , Tell R, Lau D, Parsons JR, Bush S, Perera J, . Clinical validation of the tempus xT next-generation targeted oncology sequencing assay. Oncotarget 2019;10:2384–96. 31040929 10.18632/oncotarget.26797 PMC6481324

[16] Lau D , Khare S, Stein MM, Jain P, Gao Y, BenTaieb A, . Integration of tumor extrinsic and intrinsic features associates with immunotherapy response in non-small cell lung cancer. Nat Commun 2022;13:4053. 35831288 10.1038/s41467-022-31769-4 PMC9279502

[17] Cao L , Huang C, Cui Zhou D, Hu Y, Lih TM, Savage SR, . Proteogenomic characterization of pancreatic ductal adenocarcinoma. Cell 2021;184:5031–52.e26. 34534465 10.1016/j.cell.2021.08.023 PMC8654574

[18] Cancer Genome Atlas Research Network . Integrated genomic characterization of pancreatic ductal adenocarcinoma. Cancer Cell 2017;32:185–203.e13. 28810144 10.1016/j.ccell.2017.07.007 PMC5964983

[19] Biankin AV , Waddell N, Kassahn KS, Gingras M-C, Muthuswamy LB, Johns AL, . Pancreatic cancer genomes reveal aberrations in axon guidance pathway genes. Nature 2012;491:399–405. 23103869 10.1038/nature11547 PMC3530898

[20] Waddell N , Pajic M, Patch A-M, Chang DK, Kassahn KS, Bailey P, . Whole genomes redefine the mutational landscape of pancreatic cancer. Nature 2015;518:495–501. 25719666 10.1038/nature14169 PMC4523082

[21] AACR Project GENIE Consortium . AACR Project GENIE: powering precision medicine through an international consortium. Cancer Discov 2017;7:818–31. 28572459 10.1158/2159-8290.CD-17-0151 PMC5611790

[22] Byrne KT , Betts CB, Mick R, Sivagnanam S, Bajor DL, Laheru DA, . Neoadjuvant selicrelumab, an agonist CD40 antibody, induces changes in the tumor microenvironment in patients with resectable pancreatic cancer. Clin Cancer Res 2021;27:4574–86. 34112709 10.1158/1078-0432.CCR-21-1047 PMC8667686

[23] Hewitt DB , Nissen N, Hatoum H, Musher B, Seng J, Coveler AL, . A phase 3 randomized clinical trial of chemotherapy with or without algenpantucel-L (HyperAcute-Pancreas) immunotherapy in subjects with borderline resectable or locally advanced unresectable pancreatic cancer. Ann Surg 2022;275:45–53. 33630475 10.1097/SLA.0000000000004669

[24] Kamath SD , Kalyan A, Kircher S, Nimeiri H, Fought AJ, Benson A III, . Ipilimumab and gemcitabine for advanced pancreatic cancer: a phase Ib study. Oncologist 2020;25:e808–15. 31740568 10.1634/theoncologist.2019-0473 PMC7216436

[25] Katz MHG , Petroni GR, Bauer T, Reilley MJ, Wolpin BM, Stucky C-C, . Multicenter randomized controlled trial of neoadjuvant chemoradiotherapy alone or in combination with pembrolizumab in patients with resectable or borderline resectable pancreatic adenocarcinoma. J Immunother Cancer 2023;11:e007586. 38040420 10.1136/jitc-2023-007586 PMC10693876

[26] Leidner R , Conlon K, McNeel DG, Wang-Gillam A, Gupta S, Wesolowski R, . First-in-human phase I/Ib study of NIZ985, a recombinant heterodimer of IL-15 and IL-15Rα, as a single agent and in combination with spartalizumab in patients with advanced and metastatic solid tumors. J Immunother Cancer 2023;11:e007725. 37907221 10.1136/jitc-2023-007725 PMC10619015

[27] Lin C , Verma V, Lazenby A, Ly QP, Berim LD, Schwarz JK, . Phase I/II trial of neoadjuvant oregovomab-based chemoimmunotherapy followed by stereotactic body radiotherapy and nelfinavir for locally advanced pancreatic adenocarcinoma. Am J Clin Oncol 2019;42:755–60. 31513018 10.1097/COC.0000000000000599 PMC6768754

[28] O’Hara MH , O’Reilly EM, Varadhachary G, Wolff RA, Wainberg ZA, Ko AH, . CD40 agonistic monoclonal antibody APX005M (sotigalimab) and chemotherapy, with or without nivolumab, for the treatment of metastatic pancreatic adenocarcinoma: an open-label, multicentre, phase 1b study. Lancet Oncol 2021;22:118–31. 33387490 10.1016/S1470-2045(20)30532-5

[29] O’Neill C , Hayat T, Hamm J, Healey M, Zheng Q, Li Y, . A phase 1b trial of concurrent immunotherapy and irreversible electroporation in the treatment of locally advanced pancreatic adenocarcinoma. Surgery 2020;168:610–6. 32631655 10.1016/j.surg.2020.04.057

[30] Overman M , Javle M, Davis RE, Vats P, Kumar-Sinha C, Xiao L, . Randomized phase II study of the Bruton tyrosine kinase inhibitor acalabrutinib, alone or with pembrolizumab in patients with advanced pancreatic cancer. J Immunother Cancer 2020;8:e000587. 32114502 10.1136/jitc-2020-000587 PMC7057435

[31] Padrón LJ , Maurer DM, O’Hara MH, O’Reilly EM, Wolff RA, Wainberg ZA, . Sotigalimab and/or nivolumab with chemotherapy in first-line metastatic pancreatic cancer: clinical and immunologic analyses from the randomized phase 2 PRINCE trial. Nat Med 2022;28:1167–77. 35662283 10.1038/s41591-022-01829-9 PMC9205784

[32] Parikh AR , Szabolcs A, Allen JN, Clark JW, Wo JY, Raabe M, . Radiation therapy enhances immunotherapy response in microsatellite stable colorectal and pancreatic adenocarcinoma in a phase II trial. Nat Cancer 2021;2:1124–35. 35122060 10.1038/s43018-021-00269-7 PMC8809884

[33] Reiss KA , Mick R, Teitelbaum U, O’Hara M, Schneider C, Massa R, . Niraparib plus nivolumab or niraparib plus ipilimumab in patients with platinum-sensitive advanced pancreatic cancer: a randomised, phase 1b/2 trial. Lancet Oncol 2022;23:1009–20. 35810751 10.1016/S1470-2045(22)00369-2 PMC9339497

[34] Rojas LA , Sethna Z, Soares KC, Olcese C, Pang N, Patterson E, . Personalized RNA neoantigen vaccines stimulate T cells in pancreatic cancer. Nature 2023;618:144–50. 37165196 10.1038/s41586-023-06063-y PMC10171177

[35] Wang-Gillam A , Lim K-H, McWilliams R, Suresh R, Lockhart AC, Brown A, . Defactinib, pembrolizumab, and gemcitabine in patients with advanced treatment refractory pancreatic cancer: a phase I dose escalation and expansion study. Clin Cancer Res 2022;28:5254–62. 36228156 10.1158/1078-0432.CCR-22-0308 PMC9772237

[36] Zamarin D , Hamid O, Nayak-Kapoor A, Sahebjam S, Sznol M, Collaku A, . Mogamulizumab in combination with durvalumab or tremelimumab in patients with advanced solid tumors: a phase I study. Clin Cancer Res 2020;26:4531–41. 32586937 10.1158/1078-0432.CCR-20-0328 PMC8375360

[37] Hingorani SR , Wang L, Multani AS, Combs C, Deramaudt TB, Hruban RH, . Trp53R172H and KrasG12D cooperate to promote chromosomal instability and widely metastatic pancreatic ductal adenocarcinoma in mice. Cancer Cell 2005;7:469–83. 15894267 10.1016/j.ccr.2005.04.023

[38] Ademuyiwa FO , Tao Y, Luo J, Weilbaecher K, Ma CX. Differences in the mutational landscape of triple-negative breast cancer in African Americans and Caucasians. Breast Cancer Res Treat 2017;161:491–9. 27915434 10.1007/s10549-016-4062-y PMC5243212

[39] Keenan T , Moy B, Mroz EA, Ross K, Niemierko A, Rocco JW, . Comparison of the genomic landscape between primary breast cancer in African American versus white women and the association of racial differences with tumor recurrence. J Clin Oncol 2015;33:3621–7. 26371147 10.1200/JCO.2015.62.2126 PMC4979243

[40] Whelan K , Dillon M, Strickland KC, Pothuri B, Bae-Jump V, Borden LE, . TP53 mutation and abnormal p53 expression in endometrial cancer: associations with race and outcomes. Gynecol Oncol 2023;178:44–53. 37748270 10.1016/j.ygyno.2023.09.009

[41] Williams LA , Butler EN, Sun X, Allott EH, Cohen SM, Fuller AM, . TP53 protein levels, RNA-based pathway assessment, and race among invasive breast cancer cases. NPJ Breast Cancer 2018;4:13. 29951581 10.1038/s41523-018-0067-5 PMC6018637

[42] van Beek EJAH , Hernandez JM, Goldman DA, Davis JL, McLaughlin K, Ripley RT, . Rates of TP53 mutation are significantly elevated in African American patients with gastric cancer. Ann Surg Oncol 2018;25:2027–33. 29725898 10.1245/s10434-018-6502-x PMC6644702

[43] Arora K , Tran TN, Kemel Y, Mehine M, Liu YL, Nandakumar S, . Genetic ancestry correlates with somatic differences in a real-world clinical cancer sequencing cohort. Cancer Discov 2022;12:2552–65. 36048199 10.1158/2159-8290.CD-22-0312 PMC9633436

[44] Kang M , Shen XJ, Kim S, Araujo-Perez F, Galanko JA, Martin CF, . Somatic gene mutations in African Americans may predict worse outcomes in colorectal cancer. Cancer Biomark 2013;13:359–66. 24440976 10.3233/CBM-130366 PMC4589188

[45] Myer PA , Lee JK, Madison RW, Pradhan K, Newberg JY, Isasi CR, . The genomics of colorectal cancer in populations with African and European ancestry. Cancer Discov 2022;12:1282–93. 35176763 10.1158/2159-8290.CD-21-0813 PMC9169495

[46] Hobbs GA , Baker NM, Miermont AM, Thurman RD, Pierobon M, Tran TH, . Atypical KRAS^G12R^ mutant is impaired in PI3K signaling and macropinocytosis in pancreatic cancer. Cancer Discov 2020;10:104–23. 31649109 10.1158/2159-8290.CD-19-1006 PMC6954322

[47] Wang X , Hou K, Ricciuti B, Alessi JV, Li X, Pecci F, . Additional impact of genetic ancestry over race/ethnicity to prevalence of KRAS mutations and allele-specific subtypes in non-small cell lung cancer. HGG Adv 2024;5:100320. 38902927 10.1016/j.xhgg.2024.100320 PMC11452329

[48] Lu C , Ahmed R, Lamri A, Anand SS. Use of race, ethnicity, and ancestry data in health research. PLOS Glob Public Health 2022;2:e0001060. 36962630 10.1371/journal.pgph.0001060 PMC10022242

[49] Calagua C , Russo J, Sun Y, Schaefer R, Lis R, Zhang Z, . Expression of PD-L1 in hormone-naïve and treated prostate cancer patients receiving neoadjuvant abiraterone acetate plus prednisone and leuprolide. Clin Cancer Res 2017;23:6812–22. 28893901 10.1158/1078-0432.CCR-17-0807

[50] Jiagge EM , Ulintz PJ, Wong S, McDermott SP, Fossi SI, Suhan TK, . Multiethnic PDX models predict a possible immune signature associated with TNBC of African ancestry. Breast Cancer Res Treat 2021;186:391–401. 33576900 10.1007/s10549-021-06097-8

[51] Marczyk M , Qing T, O’Meara T, Yagahoobi V, Pelekanou V, Bai Y, . Tumor immune microenvironment of self-identified African American and non-African American triple negative breast cancer. NPJ Breast Cancer 2022;8:88. 35869114 10.1038/s41523-022-00449-3 PMC9307813

[52] Sartor O , Armstrong AJ, Ahaghotu C, McLeod DG, Cooperberg MR, Penson DF, . Survival of African-American and Caucasian men after sipuleucel-T immunotherapy: outcomes from the PROCEED registry. Prostate Cancer Prostatic Dis 2020;23:517–26. 32111923 10.1038/s41391-020-0213-7 PMC7423504

[53] Unger JM , Hershman DL, Osarogiagbon RU, Gothwal A, Anand S, Dasari A, . Representativeness of Black patients in cancer clinical trials sponsored by the National Cancer Institute compared with pharmaceutical companies. JNCI Cancer Spectr 2020;4:pkaa034. 32704619 10.1093/jncics/pkaa034 PMC7368466

[54] Murthy VH , Krumholz HM, Gross CP. Participation in cancer clinical trials: race-sex-and age-based disparities. JAMA 2004;291:2720–6. 15187053 10.1001/jama.291.22.2720

[55] Pittell H , Calip GS, Pierre A, Ryals CA, Altomare I, Royce TJ, . Racial and ethnic inequities in US oncology clinical trial participation from 2017 to 2022. JAMA Netw Open 2023;6:e2322515. 37477920 10.1001/jamanetworkopen.2023.22515 PMC10362465

[56] Tejeda HA , Green SB, Trimble EL, Ford L, High JL, Ungerleider RS, . Representation of African-Americans, hispanics, and whites in national cancer Institute cancer treatment trials. J Natl Cancer Inst 1996;88:812–6. 8637047 10.1093/jnci/88.12.812

[57] Loree JM , Anand S, Dasari A, Unger JM, Gothwal A, Ellis LM, . Disparity of race reporting and representation in clinical trials leading to cancer drug approvals from 2008 to 2018. JAMA Oncol 2019;5:e191870. 31415071 10.1001/jamaoncol.2019.1870 PMC6696743

[58] Nassar AH , Adib E, Kwiatkowski DJ. Distribution of KRAS^G12C^ somatic mutations across race, sex, and cancer type. N Engl J Med 2021;384:185–7. 33497555 10.1056/NEJMc2030638

[59] Booker BD , Markt SC, Schumacher FR, Rose J, Cooper G, Selfridge JE, . Variation in KRAS/NRAS/BRAF-mutation status by age, sex, and race/ethnicity among a large cohort of patients with metastatic colorectal cancer (mCRC). J Gastrointest Cancer 2024;55:237–46. 37355486 10.1007/s12029-023-00954-z

[60] Rubin JB , Lagas JS, Broestl L, Sponagel J, Rockwell N, Rhee G, . Sex differences in cancer mechanisms. Biol Sex Differ 2020;11:17. 32295632 10.1186/s13293-020-00291-x PMC7161126

[61] Zhu C , Boutros PC. Sex differences in cancer genomes: much learned, more unknown. Endocrinology 2021;162:bqab170. 34402895 10.1210/endocr/bqab170 PMC8439393

[62] Cornelius ME , Loretan CG, Jamal A, Davis Lynn BC, Mayer M, Alcantara IC, . Tobacco product use among adults—United States, 2021. MMWR Morb Mortal Wkly Rep 2023;72:475–83. 37141154 10.15585/mmwr.mm7218a1 PMC10168602

